# Oral Health‐Related Quality of Life in Children and Adolescents With Trisomy 21 and Their Families, Associated Factors, and the Impact of Dental Interventions: A Scoping Review

**DOI:** 10.1111/scd.70237

**Published:** 2026-08-20

**Authors:** Cibelle Cristina Oliveira dos Santos, Natália Cristina Ruy Carneiro, Flavia da Costa Rosa, Ana Cristina Borges‐Oliveira, Carlos Flores‐Mir, Fernanda Signorelli Calazans, Bruna Lavinas Sayed Picciani

**Affiliations:** ^1^ Postgraduate Program in Dentistry Institute of Health of Nova Friburgo Fluminense Federal University Nova Friburgo Rio de Janeiro Brazil; ^2^ Faculty of Dentistry Faculdade Anhanguera Unidade Timbiras Belo Horizonte Minas Gerais Brazil; ^3^ Department of Community and Preventive Dentistry School of Dentistry Universidade Federal de Minas Gerais Belo Horizonte Minas Gerais Brazil; ^4^ Division of Orthodontics School of Dentistry University of Alberta Edmonton Alberta Canada; ^5^ Department of Restorative Dentistry Institute of Health of Nova Friburgo Fluminense Federal University Nova Friburgo Rio de Janeiro Brazil

**Keywords:** caregivers, down syndrome, oral health, quality of life

## Abstract

**Aims:**

To map the evidence on oral health‐related quality of life (OHRQoL) in children and adolescents with Trisomy 21 (T21) and their families, identifying associated factors and impact of dental interventions.

**Methods and Results:**

This review was registered on the Open Science Framework. Five databases (MedLine/PubMed, Web of Science, Embase, Scopus, LILACS) and the grey literature were searched, without language or year restrictions. Two independent reviewers screened records and extracted data on study characteristics, instruments, clinical conditions, and findings. Of 618 records, eight studies met the eligibility criteria. Children and adolescents with Down syndrome (T21) had a high prevalence of caries, periodontal disease, and malocclusion associated with worse OHRQoL; untreated caries, plaque, malocclusion, and poorer periodontal indices were the strongest determinants. 30%–46% of caregivers reported negative effects on their own quality of life, amid socioeconomic vulnerability and low education. Two exploratory studies with small samples and limited or no appropriate control groups suggested possible benefits of full‐mouth rehabilitation under anesthesia and orthodontic aligner treatment.

**Conclusion:**

Children and adolescents with Down syndrome bear a substantial burden of oral disease affecting OHRQoL, with possible effects on family members. Despite methodological limitations, these findings support incorporating OHRQoL assessment into routine care and developing inclusive oral health protocols.

## 1 Introduction

Trisomy 21 (T21), commonly known as Down syndrome, is the most prevalent chromosomal abnormality worldwide and is associated with multiple systemic, neurodevelopmental, and craniofacial alterations that may substantially affect oral health [1]. Children and adolescents with T21 frequently present with periodontal disease, malocclusion, delayed tooth eruption, dental anomalies, and impaired oral motor function [2, 3].These alterations increase susceptibility to oral diseases and may compromise essential daily functions such as mastication, speech, and oral hygiene, with potential consequences for physical, emotional and social well‐being [1, 4]. Oral health‐related quality of life (OHRQoL) is a multidimensional construct that reflects the impact of oral conditions on individuals daily functioning and psychosocial well‐being [5]. In populations with T21, evaluating OHRQoL is particularly relevant due to the interaction between oral diseases, intellectual disability, systemic comorbidities, and communication limitations [4].

Previous studies have shown that oral conditions such as dental caries, periodontal disease, and malocclusion may negatively affect OHRQoL in individuals with T21 [1, 2]. Moreover, the consequences of compromised oral health often extend beyond the affected individual, influencing family dynamics and caregiver well‐being by increasing emotional burden, stress, and disruptions in daily routines [1, 2, 6, 7, 8] Preliminary evidence suggests that dental interventions may improve both individual and family OHRQoL in children with T21 [5] and similar improvements in OHRQoL after dental treatment have also been reported in patients with intellectual disability [9].

Although interest in this topic has increased in recent years, the available evidence remains limited and heterogeneous. In addition, many investigations focus exclusively on either the individual with T21 or the caregiver perspective, limiting a comprehensive understanding of the broader impact of oral health on both individuals and their families [1, 3, 4].In contexts where evidence is sparse, conceptually diverse, or methodologically heterogeneous, scoping reviews are recommended as an appropriate approach to map the available literature and identify knowledge gaps [10]. Therefore, this scoping review aimed to map the available evidence on oral health–related quality of life in children and adolescents with T21 and their families, and to identify associated factors and the reported impact of dental interventions.

## 2 Materials and Methods

### 2.1 Review Design

This scoping review was conducted in accordance with the Preferred Reporting Items for Systematic Reviews and Meta‐Analyses extension for Scoping Reviews (PRISMA‐ScR) [11]. The review question was developed using the PCC framework (Population, Concept, Context). The study protocol was registered on Open Science Framework under DOI 10.17605/OSF.IO/YZE83 (available at https://osf.io/yze83/).

### 2.2 Eligibility Criteria

The PCC mnemonic (Population, Concept, and Context) [12] was employed to guide the formulation of the research question “What do we know about oral health‐related quality of life in children and adolescents with T21 and their families?”. The following elements were considered:

**Population**: Children and adolescents with T21 and their families.

**Concept**: Oral Health‐related Quality of Life

**Context**: Home and Community Environment

The inclusion criteria included studies that assessed oral health‐related quality of life in children and adolescents with T21 and their families. The exclusion criteria comprised studies that used non‐validated instruments to assess oral health‐related quality of life, studies including only participants without T21, book chapters, clinical reports, case reports, case series, literature reviews, narrative reviews, editorials, study protocols, and conference abstracts. No restrictions were applied regarding language or year of publication.

The objectives of this review include mapping the OHRQoL instruments currently in use, identifying associated factors, and assessing the impact of interventions. Therefore, validated and comparable measures are required, following JBI guidance [12]. This criterion excluded no studies, as all eligible records used validated instruments. Although the focus was on children and adolescents with T21, studies with mixed samples, including adolescents and adults, were accepted when the results were interpretable for the age range of interest, due to the scarcity of evidence on dental interventions in this population. Only one study presented a mixed sample of adolescents and adults with T21 (mean age 20.3 ± 7.4 years; range, 11.2–32.9 years), and it was the only available source on orthodontic treatment with clear aligners in individuals with T21.

### 2.3 Databases and Search Strategy

A systematic search of the literature related to oral health‐related quality of life in children and adolescents with T21 was conducted in May 2025. Alerts were set in the databases to identify any studies that might be published after the completion of the searches. Five electronic databases were searched: MedLine/PubMed; Web of science; Embase; Scopus; Lilacs. Grey literature sources were also searched through the following databases: Proquest and Google Scholar. The reference lists of included studies were also searched. The search strategy employed in PubMed (Table 1) included indexed Mesh terms and their synonyms. Then, some adjustments were made to align the strategy according to each database. Search strategies can be found in Appendix 1.

**TABLE 1:** Database and search strategy.

Database	Search strategy
PubMed	(((((((((((“Quality of life”) OR (“Life Quality”)) OR (“Quality of Life”[MeSH])) OR (“Health‐Related Quality of Life”)) OR (“Health Related Quality of Life”)) OR (“HRQOL”)) OR (“Oral‐health‐related quality of life”)) OR (“Oral health related quality of life”)) OR (“Oral health‐related quality of life”)) OR (“OHRQOL”)) AND ((((down syndrome[MeSH Terms]) OR (trisomy 21[MeSH Terms])) OR (“trisomy 21”[Title/Abstract])) OR (“down syndrome”[Title/Abstract]))) AND (((((((“Oral Health”[MeSH]) OR (“Dental Caries”[MeSH])) OR (“dental care”[MeSH Terms])) OR (“oral hygiene”[MeSH Terms])) OR (“gingivitis”)) OR (“Periodontal Diseases”)) OR (“malocclusion”))

*Note*: Adapted search strategies for Web of Science, Embase, Scopus, LILACS, ProQuest, and Google Scholar are available in Appendix 1.

All the references obtained in the search were imported to Mendeley's reference manager (Mendeley Desktop Software; V‐1.17.10) for duplicate removal. Then, the references were merged in the web software Rayyan (Qatar Computing Research Institute, Doha, Qatar), a mobile app for systematic reviews [13] to continue the selection phases.

### 2.4 Selection of Studies

Records exported to Rayyan were screened independently and blindly by two reviewers (XX and XX). Titles and abstracts were assessed according to the predefined eligibility criteria and classified as included, maybe, or excluded. Disagreements were resolved through discussion. In the second stage, the same reviewers independently assessed the full texts of potentially eligible studies. Any remaining disagreements were resolved by discussion or, when necessary, by consultation with a third reviewer (XX) until consensus was reached.

### 2.5 Data Extraction

For the included studies, two reviewers independently extracted data from the full texts using a customized extraction form. The following information was collected: authors and year of publication, country, aim, study design, sample size, age, sex, oral health‐related quality of life instrument, results, main conclusions, and reported recommendations or gaps in the literature. Disagreements were resolved through discussion. When important data were unclear or missing, the corresponding authors were contacted by e‐mail. If no response was obtained, unavailable data were reported in Table 2 as missing or not provided.

**TABLE 2:** Summary data of included studies.

		3. Population Caregivers	Individuals with					
1. Authorship Year of publication, country study design	2. Objective	‐N (Male/Female) ‐Age	T21 ‐N (M/F) ‐Age ‐Prevalence of comorbidities	4. Concept ‐Quality of life assessment tools ‐Statistical analysis	5. Context ‐Economic situation ‐Caregiver occupation ‐Caregiver's education ‐ Oral health conditions of individuals with T21 ‐Use of dental services	6. Outcomes	7. Main conclusion	8.Other informations ‐Gaps in literature ‐Limitations of the study
**Aljameel et al., 2021, Saudi Arabia, observational cross‐sectional study**.	To evaluate the impact of OHRQoL of children/adolescents with T21 and their families.	−63 (F) −34 to 58 years.	−63 (27/36) −10 to 14 years. −85.7%	OH‐QOLADS applied to caregivers and individuals with T21. −Spearman's correlation, Kruskal‐Wallis, and Mann‐Whitney tests.	−N.I −82% unemployed −13% with no schooling, 57% with primary to secondary education, 30% with university education −25.4% with good oral health, 46.7% with fair oral health and 27% with poor oral health −N.I	−34.9% of parents reported a negative impact of oral health on their children's quality of life, with 54% experiencing pain. −46% indicated that the child/adolescent's oral health affected their quality of life −Worse perceived oral health in children was significantly correlated with higher OHRQoL scores, reflecting greater family impact (*p* < 0.001).	‐Inadequate oral health has significant negative impacts on various aspects of children's lives as well as those of their families.	‐Cross‐sectional design and reliance on self‐reported data.
**Carrada et al., 2019, Brazil, observational cross‐sectional study**.	To evaluate the impact of oral health conditions in children and adolescents with T21 on their families’ quality of life, in comparison with a control group without T21.	−144 (primarily woman) N.I	−144 (77/67) −9.01 years (± 3.72). −54.2%	−FIS. −Pearson's correlation, Fisher's exact test, Wilcoxon test, and Poisson regression.	−The majority have low educational levels and vulnerable socioeconomic conditions. −41.3% have dental caries, 59.8% malocclusion, with 31.5% classified as severe and 45.7% periodontal disease. −Only 2.2% undergoes orthodontic treatment. −Most have received some dental treatment; however, the frequency of specialized care is low	−No difference (*p* > 0.05) was found between groups with and without T21 −Dental caries increased caregivers’ negative perception (PR = 3.95; *p* < 0.01). Untreated caries consequences raised the impact 1.83 times (*p* = 0.01). Defined malocclusion was associated with a 2.75‐fold increase (*p* = 0.01) in negative impact, while severe malocclusion resulted in a 2.82‐fold increase (*p* = 0.04).	−Dental caries, untreated caries, and malocclusion negatively affected the OHRQoL of families of children/adolescents with T21. −However, this impact was not greater than in the control group.	−Few studies have used standardized measurement instruments. −Cross‐sectional design and limited representativeness.
**Carrada et al., 2020, Brazil, observational cross‐sectional study**.	To evaluate caregivers’ perceptions of the impact of oral health conditions on the OHRQoL of their children with T21, and to compare these perceptions with those of caregivers of children without T21.	−144 (primarily woman). −26 to 64 years.	−144 (77/67) −9.01 years (± 3.72). −54.2% congenital heart disease: 31.2%, respiratory: 18.7%, and endocrine: 14.6%.	−P‐CPQ −Wilcoxon, Qui‐square, Fisher and Poisson regression.	−29.2% with income up to 5 minimum wages, 70.8% with income above 5 minimum wages −N.I −69.4% of mothers had ≤ 8 years of education, 0.6% of mothers had > 8 years of education. −Dental caries: 64.6%, gingival bleeding: 71.2%, dental calculus: 82.9% and periodontal pockets: 36.9% −Need for dental treatment: 81.1% of participants −20% had never visited a dentist.	−Parents of children/ adolescents with T21 had a more negative perception of the impact of oral health conditions on their children's quality of life, especially in the domain of functional limitations and in the total P‐CPQ score (*p* < 0.05). −Clinical consequences of untreated caries increased the likelihood of negative perception by 1.72 times. −The presence of visible plaque increased the likelihood of negative perception by 1.48 times.	−Caregivers of individuals with T21 reported more negative perceptions of the impact of oral health conditions on their children's quality of life compared with parents of children without T21. −Clinical consequences of untreated caries and the presence of visible plaque were the main determinants of this negative perception.	‐There is a lack of comparative studies with control groups without T21, and most available data focus on periodontal disease rather than caries or malocclusion. ‐Few studies use validated instruments, relying mainly on caregiver perception.
**Essam, et al., 2023, Egypt, observational cross‐sectional study**.	To assess parental perception of OHRQoL in children and adolescents with T21.	−194 (16/178) −38.61 years (± 7.25)	−**≅** 194 (N.I) −6.46 years (± 2.38) −N.I	−Allison and Lawrence questionnaire (2005) applied to caregivers. −*t*‐ test.	−Lower socioeconomic status −91.8% of mothers and 9.8% of fathers were unemployed. −8.8% of fathers were illiterate, and 49.5% had completed high school. −36.6% of mothers had secondary education, while 13.9% were illiterate. −53.6% had good oral health, 30.4% fair, 13.4% poor and 2.6% excellent. −N.I	−80.9% of parents reported a positive perception of their children's oral health and a favorable impact on their OHRQoL. −Older parents reported lower OHRQoL scores (*p* = 0.016). −Families with more than five children had higher scores for perceived problems or impacts on OHRQoL (*p* = 0.016). −Parents with lower educational levels reported higher perceived impact on quality of life (*p* = 0.011).	‐Although parental perception of OHRQoL is mostly positive, there is a lack of guidance provided to caregivers, highlighting the need to improve oral health education and access to information and specialized care for children with T21.	‐Few studies assess the impact of oral health problems on the quality of life of individuals with T21 or how sociodemographic factors influence this perception. ‐Existing research is limited by cross‐sectional design, small sample sizes, reliance on self‐reported questionnaires.
**Loureiro et al., 2007, Brazil, observational, cross‐sectional study**.	To determine the prevalence of periodontal disease in children/adolescents with T21 and its impact on their quality of life.	−93 (F) −N.I.	−93 (N.I) −6 to 20 years. −N.I.	− OHIP‐14 −Kruskal‐Wallis and Mann‐Whitney.	−N.I −N.I. −N.I. −68% T21 had bad or extremely bad oral hygiene; 91% had bleeding on probing; 33% of the female had probing depth ≥ 4 mm; 25% of male had probing depth ≥ 4 mm; 30% present the level of attachment loss from 4 to 6 mm and in 10% it was > 6 mm. −NI.	‐Positive correlation between clinical parameters (bleeding, depth, attachment loss) and QoL scores (*p* < 0.001).	‐Periodontal disease can be considered as a condition with high prevalence with T21 children and adolescents and it has a great impact in their quality of life.	−The results of the study might reflect the expectation of mothers in relation to the son's/daughter's appearance. −The reports of parents might not fully correspond to what is felt by their son's/daughter's.
**Mohamed et al., 2024, Egypt, Before‐and‐after observational intervention study**.	‐To assess OHRQOL after full mouth rehabilitation under general anesthesia in Egyptian children with T21.	−30 (N.I) −N.I	−30 (16/14) −6.79 years (± 2.34).	−ECOHIS −Family Impact Scale −N.I	−N.I − N.I −N.I − N.I −N.I	−Improvement in FIS overall score (1.98→1.16, *p* < 0.001). −Improvement in ECOHIS overall score (2.18→1.66, *p* < 0.001)	−OHRQoL improved significantly after mouth rehabilitation in overall aspects, child symptoms, child interaction, child psychology and child self‐image. −There was no impact in family financials, parental stress, and child function.	‐Long‐term follow‐up is need with a larger sample size and control group.
**Sesiliana et al., 2021, Observational** **Study**.	To determine and evaluate the relationship between parenting stress in parents with OHRQoL in children with T21.	−33 (female) −Age: 31–40 years = 13 (39.4%) 41–50 years = 16 (48.5%) 50 years = 4 (12.1%)	−33 (24/9) −2‐18 years = 31 (93.94%) > 18 years = 2 (6.06%) − N.I	−ECOHIS −Descriptive statistics.	−N.I −Employed [12 (36%)] Not employed [21 (63.64%)] −Caregiver's education: High school = 10 (30.30%) Diploma = 16 (18.18%) Bachelor = 17 (51.52%) −N.I −N.I	−57.6% of children showed low ECOHIS impact, while 42.4% had moderate/high impact. − The child domain was quite impacted (37.97%), with pain the key driver (84.85%), affecting functions and psychosocial aspects. −The family domain was lower (30.99%).	−About 40% of children with Down syndrome show moderate/high impact on OHRQoL, concentrated in the child domain, with pain as the main driver of functional and psychosocial losses. −The family impact is lower but clinically relevant (time, costs, schedule disruptions).	−No analysis and hypothesis testing on the causal relationship between individual and external factors and parenting stress. − No analysis of the impact of demographic variables and clinical examination of the teeth and mouth on OHRQoL in children with T21.
**Taniguchi et al., 2024, Brazil,** **Controlled clinical trial study**	To assess the impact of treatment with orthodontic aligners (OA) on oral health‐related quality of life (OHRQoL) in patients with T21 compared to non‐syndromic patients.	−N.I −N.I	−10 (7/3) −20.3 years (± 7.4)	‐Oral Health Scale for People with Down's syndrome (OHDS) ‐Brazilian version of the OHIP‐14 ‐Friedman and Mann‐Whitney tests.	‐N.I ‐N.I ‐N.I ‐N.I ‐N.I	‐OHDS: After 12 months, there was a significant reduction in the total score, indicating a lower impact on OHRQoL. Improvements were seen in. ‐OHIP‐14: At baseline, the T21 group had greater functional limitation than controls (*p* < .001). Over 12 months, T21 showed significant reductions in physical disability, indicating improved OHRQoL. The control group showed no changes across time points.	‐The treatment with orthodontic aligners positively impacted OHRQoL of T21 patients, and these results were perceived by caregivers, mainly in relation to issues related to eating and communication.	‐The OHIP‐14 is self‐administered instrument intended to be completed by participants. However, some T21 patients required assistance to respond. ‐Few patients included in this study because of the difficulty in obtaining a sample (*n* = 10).

Abbreviations: ECOHIS, Early Childhood Oral Health Impact Scale; F, female; FIS, Family Impact Scale; M, male; N.I, not informed; OHDS, Oral Health Scale for People with Down Syndrome; OHIP‐14, Oral Health Impact Profile–14; OH‐QOLADS, Oral Health–Related Quality of Life for Children with Down Syndrome; P‐CPQ, Parental‐Caregiver Perceptions Questionnaire;.

## 3 Results

### 3.1 Selection of Sources of Evidence

**FIGURE 1:**
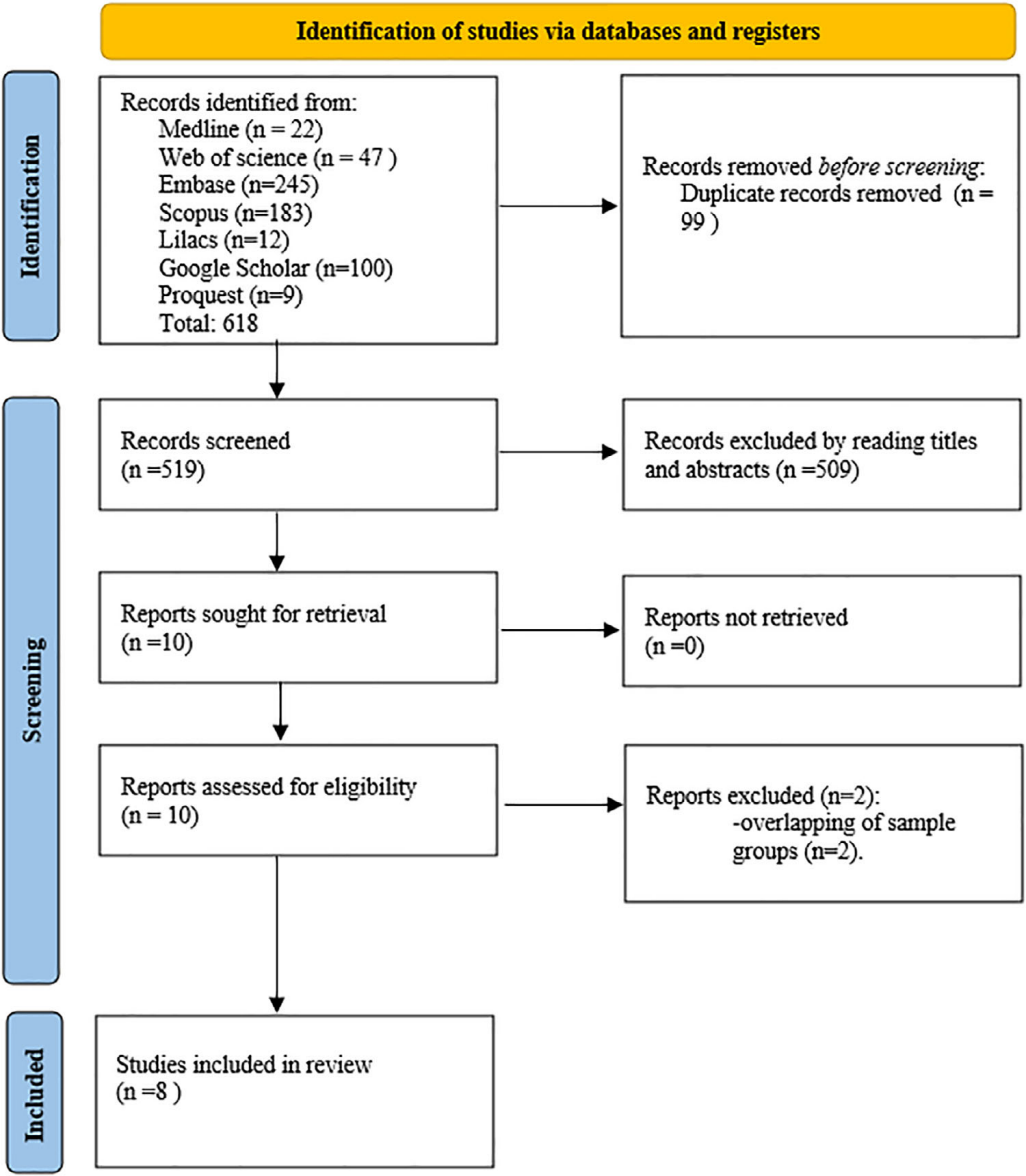
Flow diagram of study identification.

A total of 618 records were identified through database searches. After removal of 99 duplicates, 519 titles and abstracts were screened, and 509 records were excluded. Ten full‐text studies were assessed for eligibility, and two were excluded because of overlapping samples. Therefore, eight studies were included in this review [1, 2, 5, 6, 7, 14, 15, 16]. The selection process is depicted in the PRISMA‐ScR flow diagram (Figure 1).

### 3.2 Characteristics of Sources of Evidence

All eight included articles were available in English between 2007 and 2024. Four were conducted in Brazil, [2, 5, 7, 16] one in Saudi Arabia [1], one in Indonesia [15], and two in Egypt [6, 14]. One study was a controlled clinical study, one was a before‐and‐after observational intervention study, and the remaining six were cross‐sectional observational studies. Mean ages generally ranged from early childhood to adolescence, while one study included a mixed sample of adolescents and adults with T21, with a mean age of 20.3 ± 7.4 years and a range of 11.2–32.9 years (Table 2). To assess the impact of oral health on quality of life, the studies used the following instruments: OH‐QOLADS, applied to caregivers and individuals with T21 [1]; the Family Impact Scale (FIS) [7]; the Parental‐Caregiver Perceptions Questionnaire (P‐CPQ) [16]; the Allison and Lawrence questionnaire [14]; the Oral Health Impact Profile—14 items (OHIP‐14) [2, 5]; the Early Childhood Oral Health Impact Scale (ECOHIS) [6, 15]; and the Oral Health Scale for People with Down's Syndrome (OHDS) [4], according to Table 3.

**TABLE 3:** Instruments used to assess oral health–Related quality of life (OHRQoL) and their target of evaluation (child/adolescent with T21 vs caregiver/family).

Instrument	Primary OHRQoL target	Respondent	Studies using the instrument (first author, year)
**OH‐QOLADS**	Child/adolescent with T21 and family impact	Individual with T21 (with caregiver support if needed) and caregiver	Aljameel, 2021
**OHDS**	Individual with T21	Individual with T21	Taniguchi, 2024
**OHIP‐14**	Individual with T21	Individual with T21 / caregiver, depending on the study	Taniguchi, 2024; Loureiro, 2007
**ECOHIS**	Child with T21	Caregiver	Mohamed, 2024; Sesiliana, 2021
**P‐CPQ**	Child/adolescent with T21	Caregiver	Carrada, 2020
**FIS (Family Impact Scale)**	Caregiver/family quality of life	Caregiver/family	Carrada, 2019; Mohamed, 2024
**Allison & Lawrence (2005)**	Caregiver/family quality of life	Caregiver	Essam, 2023

### 3.3 Results of Individual Sources of Evidence

The eight included studies point to three central conclusions. Children and adolescents with T21 present a high prevalence of oral diseases, especially caries, periodontal disease, and malocclusion. Poorer oral conditions are systematically associated with worse OHRQoL scores, regardless of the instrument or respondent. Family impact is variable and appears to depend more on the child's oral conditions and socioeconomic context than on T21 itself. The main divergences between studies relate to the magnitude of family impact, the age profile of the samples, and the type of instrument used, which may explain the heterogeneity of the results. The detailed characteristics, methods, and key findings of each of the eight included studies are presented individually in Table 2.

### 3.4 General and Oral Health Conditions of Individuals with T21

**FIGURE 2:**
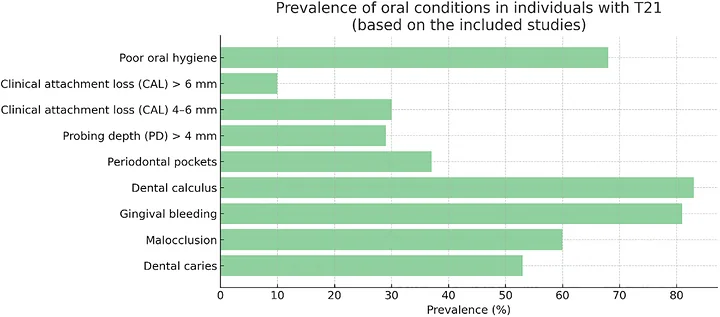
Prevalence of oral conditions in individuals with T21, based on the studies included in this scoping review.

Systemic comorbidities were highly prevalent in children and adolescents with T21, affecting up to 85.7% of participants in one study [1], mainly congenital heart disease (31%), respiratory conditions (19%), and endocrine disorders (15%) [14]. Oral health conditions were similarly prevalent across five studies (Figure 2) [1, 2, 6, 12, 14] even where samples were classified as having “good oral health” (25% [1] to 54% [12] of participants), a notable proportion remained in “fair” or “poor” condition. Dental caries ranged from 41% to 65% [6, 14], malocclusion affected about 60% of individuals (32% severe) [6], and periodontal findings included gingival bleeding in 71% to 91% [2, 14], calculus in approximately 83%, pockets in 37%, probing depths >4 mm in 25%–33%, and attachment loss of 4–6 mm in 30% or >6 mm in 10%;[2,14] inadequate oral hygiene was observed in approximately 68% of individuals [2]. Despite this burden, use of dental services remained limited: only 2.2% of participants in one study were undergoing orthodontic treatment [6], and although 81.1% needed dental treatment in another, 20% had never visited a dentist [14].

### 3.5 Impact on Individual Oral Health‐Related Quality of Life

**FIGURE 3:**
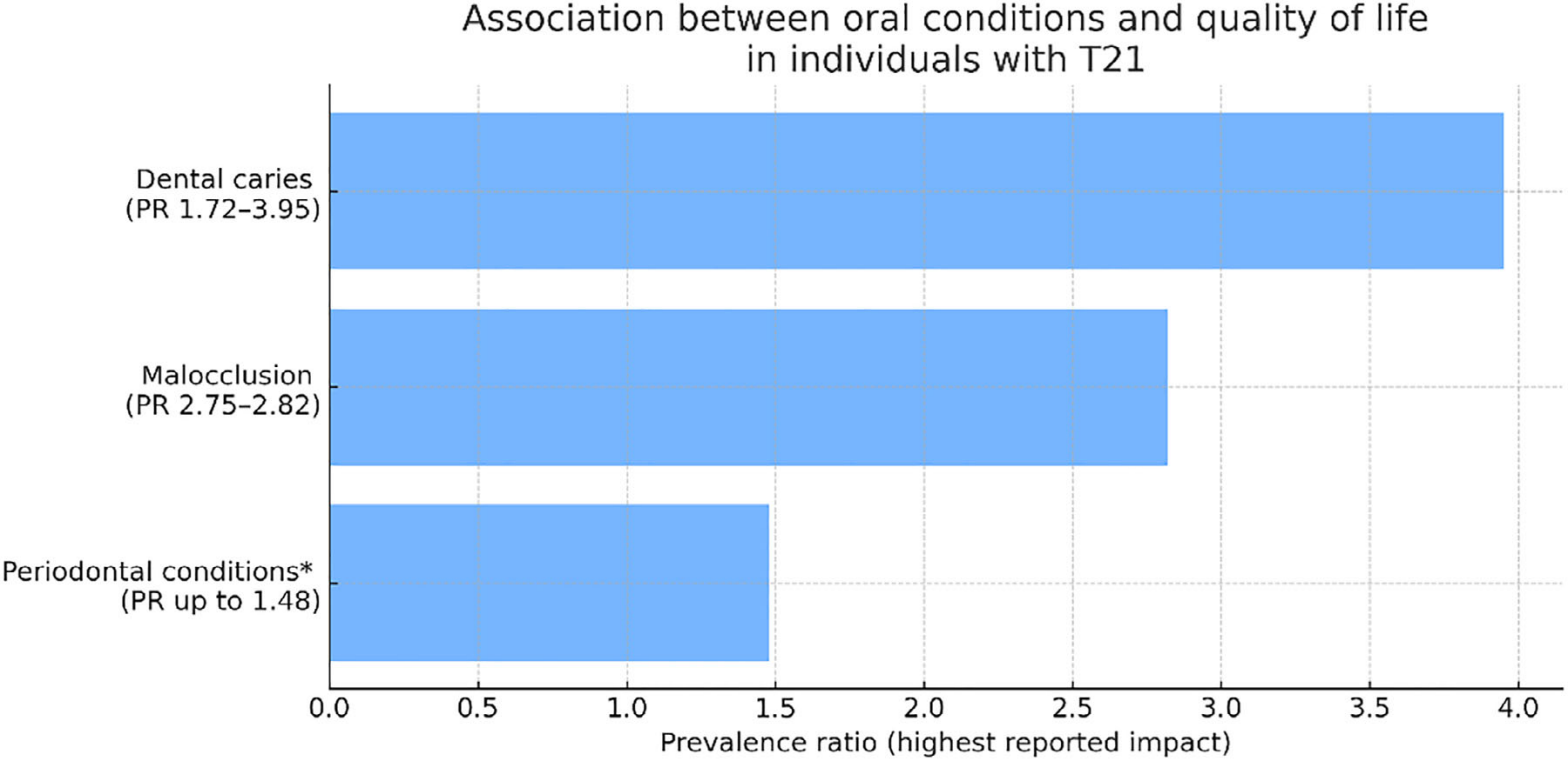
Association between oral conditions and quality of life in individuals with T21.

The seven instruments used (OH‐QOLADS, OHIP‐14, ECOHIS, P‐CPQ, FIS, and Allison & Lawrence) assessed OHRQoL from different perspectives (Table 3), but the pattern of association was convergent: poorer oral conditions translated into worse OHRQoL scores. Untreated caries (PR = 1.72), visible plaque (PR = 1.48), defined or severe malocclusion (PR = 2.75–2.82), and poorer periodontal indices (*p* < 0.001) emerged as the main determinants of negative impact (Figure 3).

### 3.6 Family/Caregiver Impact

Of the four studies that assessed family impact, three reported a negative impact, with 30% to 46% of caregivers affected, expressed mainly as frustration, worry, self‐blame, family conflict, and sleep disturbance. In contrast, the only study that included a control group without T21 found no significant difference in family impact between groups, but showed that caries (PR = 3.95), sequelae of untreated caries (PR = 1.83), and malocclusion (PR = 2.75–2.82) amplified the impact regardless of T21 status [6].

### 3.7 Social Determinants and Impact of Dental Interventions

In seven of the eight studies, caregivers were predominantly mothers facing socioeconomic vulnerability and low educational attainment, with maternal unemployment ranging from 63.6% to 91.8%. Approximately 13% to 14% of mothers had no formal education [1, 12], while 36.6% to 69.4% had basic to intermediate education, corresponding to up to 8 years of schooling [12, 14]. Finally, the proportion of mothers with a completed higher education degree varied widely across studies, ranging from 0.6% to 51.52% [13, 14].

Regarding two studies assessed the effect of dental interventions on OHRQoL, after comprehensive dental rehabilitation under general anesthesia (*n* = 30), the total FIS score decreased from 1.98 to 1.16 and the ECOHIS score from 2.18 to 1.66 (both *p* < 0.001) [6]. After 12 months of orthodontic treatment with clear aligners, the physical disability domain of the OHDS decreased (T0> T3; *p* = 0.021), and the total OHDS score was reduced by half, from 33 to 16.5 (*p* = 0.028) [5].

## 4 Discussion

Although the included studies were heterogeneous in design, instruments, and sample characteristics, they consistently indicate that oral diseases are highly prevalent in individuals with T21 and are associated with poorer oral health‐related quality of life (OHRQoL), with socioeconomic vulnerability also shaping perceived impact [1, 2, 4, 5, 6, 12, 13, 14]. Family impact was reported in most studies that assessed this outcome, mainly reflecting caregiver burden related to the child's oral conditions. However, the only study with a control group found no significant difference between families of individuals with and without T21, suggesting that oral conditions such as caries and malocclusion may be more directly related to family impact than T21 itself.

### 4.1 Strength of the Evidence Base and Generalizability

The small evidence base limits the strength and generalizability of the conclusions. The eight included studies comprised 711 individuals with T21, with heterogeneous designs, instruments, and sample sizes, which limits the precision of prevalence and impact estimates. Geographic generalizability is also limited, as half of the studies were conducted in Brazil and the remaining studies came from only three other countries. Therefore, the findings should be interpreted as evidence for research priorities and clinical hypotheses rather than as firm causal inferences or population‐specific guidelines.

### 4.2 Instrument Heterogeneity and Reliance on Proxy Reports

The seven instruments used across the eight studies differed substantially in construct assessed, respondent type, recall period, and population specificity. Some instruments focused on the individual's OHRQoL, others on family impact, and some combined both perspectives, while most were not specifically developed for individuals with intellectual disability or T21. These differences may affect sensitivity to detect impact, limit direct comparison of scores across studies, and help explain discrepancies in the magnitude of reported individual and family effects. Together, this heterogeneity supports an exploratory rather than quantitative synthesis and highlights the need for validated, population‐specific instruments.

Equally relevant is the strong reliance on caregiver reports. In six of the eight studies, caregiver report was the primary or only source of OHRQoL assessment. Although partly justified by intellectual disability and verbal communication limitations in some individuals with T21, proxy reporting may introduce bias. Caregivers may be more likely to identify observable symptoms, such as pain, functional difficulties, or gingival bleeding, while subjective and psychosocial dimensions, such as embarrassment or social repercussions, may be less accurately captured. Caregiver reports may also be influenced by the emotional, logistical, and financial burden associated with care. Since many individuals with T21, especially adolescents, may retain some capacity for assisted self‐report, future studies should consider combining adapted or pictographic instruments with parallel self‐ and proxy‐reported measures.

### 4.3 Burden of Oral Diseases, Determinants, and Impact on OHRQoL

The findings indicate a high burden of oral diseases in individuals with T21, including biofilm accumulation, caries, periodontal disease, and malocclusion, resulting from the interaction between biological and contextual vulnerability. Muscular hypotonia, craniofacial alterations, especially maxillary constriction, and the higher prevalence of Class III malocclusion and crossbite may favor plaque retention and impair mastication, while immune dysregulation and periodontopathogenic bacteria contribute to earlier and more severe periodontal disease [16, 17, 18, 19]. These factors are further amplified by limited fine motor skills, dependence on caregivers for biofilm control, and socioeconomic vulnerability, which may shape access to care and oral health priorities in families already burdened by comorbidities [20]. This disease burden is reflected in poorer OHRQoL. Untreated caries, often associated with pain, functional limitation, sleep disturbance, and behavioral changes, appears to be a major determinant of negative impact, particularly when pain recognition is delayed in individuals with communication or cognitive difficulties. Malocclusion may impair mastication, speech, and facial aesthetics, while periodontal disease, including gingival bleeding, increased probing depth, and attachment loss, has been associated with worse quality‐of‐life scores [2].

### 4.4 Family Impact

Most studies that assessed family impact reported a negative effect, affecting 30.99% to 46% of caregivers, mainly associated with the child's oral conditions [1, 5, 13]. The only study with a control group found no significant difference between families of individuals with and without T21, but showed that caries, sequelae of untreated caries, and malocclusion increased family impact regardless of T21 status [6]. This suggests that family burden may be driven more by the severity of oral conditions than by the diagnosis itself. Differences in instruments, sociocultural context, and the high prevalence of systemic comorbidities in T21 may also help explain the variability in reported impact across studies.

### 4.5 Interpretation of Intervention Effects

Two studies assessed the effect of dental interventions on OHRQoL in individuals with T21. The results of comprehensive rehabilitation under general anesthesia, although statistically significant, involved a small sample (*n* = 30), no control group, and modest changes (FIS 1.98→1.16; ECOHIS 2.18→1.66), constituting an exploratory signal rather than evidence of clinically meaningful benefit [5]. Similarly, a single 12‐month study without a matched control group suggested a positive effect of orthodontic treatment with clear aligners on perceived OHRQoL [4]. This finding is consistent with what would be expected for this modality, but given the weak design and small sample, it should be treated as a hypothesis for controlled studies, not as established evidence of a protective effect of early intervention.

### 4.6 Clinical Implications

Despite the limitations of the evidence base, the findings support a preventive, accessible, and multidisciplinary model of oral care for children and adolescents with T21. Preventive care should begin in early infancy and include caregiver guidance for biofilm control adapted to hypotonia and motor limitations, fluoride varnish, sealants when indicated, dietary counselling, shorter recall intervals, and early orthodontic assessment, given the impact of malocclusion on OHRQoL. At the service level, the gap between clinical need and access should be addressed by training general dentists and primary care teams in T21 oral management, integrating oral health into routine T21 follow‐up programs, and actively reaching vulnerable families through social work and family health teams. Given the high prevalence of systemic comorbidities, dental care should also be coordinated with pediatricians, cardiologists, endocrinologists, respiratory/therapy professionals, and psychology/social work teams, especially before procedures under general anesthesia and when sensory or behavioral management is required.

### 4.7 Limitations

Several limitations warrant attention. The small number of eligible studies (*n* = 8) and concentrated geographic distribution constrain generalizability. The predominance of cross‐sectional, often underpowered designs restricts causal inference and precludes assessing change over time. Only one study included a control group without T21, limiting the ability to distinguish syndrome‐ from condition‐attributable impact. Finally, heterogeneity in instruments and respondent type, both influenced by socioeconomic determinants, support cautious interpretation and the need for multicenter longitudinal studies with validated instruments and adequate comparison groups.

## 5 Conclusion

- Children and adolescents with T21 have a high burden of dental caries, periodontal disease, and malocclusion, which is associated with poorer oral health‐related quality of life (OHRQoL).
- Family impact is variable and appears to be influenced by the instrument used, respondent type, and sociocultural context.
- Access barriers and systemic comorbidities may further worsen this scenario.
- Although the two intervention studies suggest a potential benefit of comprehensive dental rehabilitation under general anesthesia and orthodontic treatment with clear aligners, this evidence remains preliminary and requires confirmation in controlled studies with larger samples.

## Author Contributions

**Cibelle Cristina Oliveira dos Santos**: conceptualization, methodology, investigation, data curation, writing – original draft, writing – review and editing. **Natália Cristina Ruy Carneiro**: methodology, investigation, data curation, writing – review and editing. **Flavia da Costa Rosa**: methodology, investigation, data curation, writing – review and editing. **Ana Cristina Borges‐Oliveira**: methodology, supervision, writing – review and editing. **Carlos Flores‐Mir**: methodology, supervision, writing – review and editing. **Fernanda Signorelli Calazans**: methodology, supervision, writing – review and editing. **Bruna Lavinas Sayed Picciani**: conceptualization, methodology, supervision, writing – review and editing. All authors have read and approved the final version of the manuscript.

## Funding

This study was financed in part by the Coordenação de Aperfeiçoamento de Pessoal de Nível Superior—Brasil (CAPES)—Finance Code 001.

## Conflicts of Interest

The authors declare no conflict of interest.

## Supporting information




**Supporting File 1**: scd70237‐sup‐0001‐SuppMat.docx.
