# Supplementary material for: Oral Health‐Related Quality of Life in Children and Adolescents With Trisomy 21 and Their Families, Associated Factors, and the Impact of Dental Interventions: A Scoping Review
Source: Spec Care Dentist. 2026 Aug 20;46(4):e70237. doi: 10.1111/scd.70237 (PMC13491573; doi:10.1111/scd.70237)
Supplement: Supplementary file 1 [file SCD-46-0-s001.docx]

**Appendix 1. Search strategy and databases**

| Database | Query | Items found |
| --- | --- | --- |
| Pubmed | ((((((((((("Quality of life") OR ("Life Quality")) OR ("Quality of Life"[MeSH])) OR ("Health-Related Quality of Life")) OR ("Health Related Quality of Life")) OR ("HRQOL")) OR ("Oral-health-related quality of life")) OR ("Oral health related quality of life")) OR ("Oral health-related quality of life")) OR ("OHRQOL")) AND ((((down syndrome[MeSH Terms]) OR (trisomy 21[MeSH Terms])) OR ("trisomy 21"[Title/Abstract])) OR ("down syndrome"[Title/Abstract]))) AND ((((((("Oral Health"[MeSH]) OR ("Dental Caries"[MeSH])) OR ("dental care"[MeSH Terms])) OR ("oral hygiene"[MeSH Terms])) OR ("gingivitis")) OR ("Periodontal Diseases")) OR ("malocclusion")) | **22** |
| WOS | ((((((((("Quality of life") OR ("Life Quality")) OR ("Quality of Life"[MeSH])) OR ("Health-Related Quality of Life")) OR ("Health Related Quality of Life")) OR ("HRQOL")) OR ("Oral-health-related quality of life")) OR ("Oral health related quality of life")) OR ("Oral health-related quality of life")) OR ("OHRQOL") AND (((ALL=("trisomy 21")) OR ALL=("down syndrome")) OR ALL=("Down's syndrome")) OR ALL=("Downs syndrome") AND ((((((ALL=("Oral Health")) OR ALL=("Dental Caries")) OR ALL=("dental care")) OR ALL=("oral hygiene")) OR ALL=("gingivitis")) OR ALL=("Periodontal Diseases")) OR ALL=("malocclusion")  https://www.webofscience.com/wos/woscc/summary/93ff6062-44fd-48bb-9efa-1c4a92a56cc9-0163f1ad81/relevance/1 | **47** |
| Embase | 'quality of life'/exp OR 'quality of life' OR 'life near/2 quality' OR 'health-related quality of life'/exp OR 'health-related quality of life' OR 'health related quality of life'/exp OR 'health related quality of life' OR 'hrqol' OR 'oral-health-related quality of life' OR 'oral health related quality of life'/exp OR 'oral health related quality of life' OR 'oral health-related quality of life' OR 'ohrqol' AND 'down syndrome'/exp OR 'down syndrome' OR 'trysomy 21' OR 'downs syndrome'/exp OR 'downs syndrome' AND 'oral health'/exp OR 'oral health' OR 'dental caries'/exp OR 'dental caries' OR 'dental care'/exp OR 'dental care' OR 'mouth hygiene'/exp OR 'mouth hygiene' OR 'oral hygiene'/exp OR 'oral hygiene' OR 'gingivitis'/exp OR 'gingivitis' OR 'periodontal disease'/exp OR 'periodontal disease' OR 'malocclusion'/exp OR 'malocclusion' | **245** |
| Scopus | "Quality of life" OR "Life Quality" OR "Health-Related Quality of Life" OR "Health Related Quality of Life" OR "HRQOL" OR "Oral-health-related quality of life" OR "Oral health related quality of life" OR "Oral health-related quality of life" OR "OHRQOL" AND "Oral Health" OR "Dental Caries" OR "dental care" OR "oral hygiene" OR "gingivitis" OR "Periodontal Diseases" "malocclusion" AND "down syndrome" OR "down syndrome" OR "trysomy 21" OR "down's syndrome" OR "downs syndrome" | **183** |
| Lilacs | (qualidade de vida) OR (calidad de vida) OR (qualidade de vida relacionada à saúde) OR (calidad de vida relacionada con la salud) OR (qvrs) OR (cvrs) OR (hrqol) OR (qualidade de vida relacionada a saúde bucal) OR (calidad de vida relacionada com la salud bucal) AND (síndrome de down) OR (trissomia do 21) OR (trissomia do cromossomo 21) AND (saúde bucal) OR (salud bucal) OR (cárie dental) OR (caries dentales) OR (cáries) OR (caries) OR (atenção odontológica) OR (atención odontológica) OR (higiene bucal) OR (higiene dentária) OR (higiene dental) OR (gingivitis) OR (gengivite) OR (doenças periodontais) OR (enfermedades periodontales) OR (má oclusão) OR (maloclusão) OR (maloclusión) OR (mala oclusión) | **12** |
| Google Scholar | "Oral health related quality of life" AND "down syndrome" AND "oral health" | 100 |
| Proquest | ((TS=(((((((((("Quality of life") OR ("Life Quality")) OR ("Quality of Life"[MeSH])) OR ("Health-Related Quality of Life")) OR ("Health Related Quality of Life")) OR ("HRQOL")) OR ("Oral-health-related quality of life")) OR ("Oral health related quality of life")) OR ("Oral health-related quality of life")) OR ("OHRQOL"))) AND TS=((((("trisomy 21")) OR ("down syndrome")) OR ("Down's syndrome")) OR ("Downs syndrome") )) AND TS=(((((((("Oral Health")) OR ("Dental Caries")) OR ("dental care")) OR ("oral hygiene")) OR ("gingivitis")) OR ("Periodontal Diseases")) OR ("malocclusion")) | **9** |
